# On the Nature and Structural Characteristics of Cancer, and of Those Morbid Growths Which May Be Confounded with It

**Published:** 1840

**Authors:** 


On the Nature and Structural Characteristics of Cancer, and of those Morbid Growths which may be confounded with it. By *J. Müller*, M.D. Professor of Anatomy and Physiology in the University of Berlin, &c. &c. Translated from the German, with Notes, by *Charles West*, M.D. Graduate in Medicine of the University of Berlin. Illustrated with numerous Steel Plates and Wood Engravings. Part I. Octavo, pp. 182. London, Sherwood & Co. 1840.

On the reputation of Professor Müller as a profound physiologist, we need not speak. He has united the laborious accuracy of the German with the common-sense and matter-of-fact mind of the Englishman. Such a man must carry to the department of pathology a spirit of investigation eminently calculated to achieve great results, at least if any such can be obtained by careful observation and a sound judgment.

It must be owned that the intimate nature of cancer and of the morbid growths which may be confounded with it, is at once an inviting and a forbidding subject—inviting, because it is a sort of El Dorado of pathological adventure—forbidding, because so many inquirers have made shipwreck of their labours on its confines. Will this new voyager share their fortunes?

### 1. *Uncertain External Characters of Morbid Growths.*

Professor Müller begins by some observations on the uncertainty which has attended, still attends, and, we fear, will continue to attend, the diagnosis of tumors, founded on their external characters, or on their internal appearances. So numerous, he justly observes, are the transitions into each other of those general mechanical differences which the exterior or interior of morbid growths presents, that the observation of the above-mentioned characteristics is alone insufficient for the purposes of accurate diagnosis. Growths, indeed, which are peculiar to certain tissues may often be distinguished with certainty without minute examination, as is the case with neuroma in nerves, and with polypi in mucous membranes. If, however, any organic tissue is subject to many and various changes, some of which are peculiar to it, while others occur also in different tissues, then does accurate diagnosis often become exceedingly difficult, or, in the present state of our knowledge, even impossible. These difficulties are greatest in the case of tumors which do not owe their existence to the peculiarities of any particular structure, but which may occur at the same time in many and very different tissues. A tumor of a non-malignant nature may readily assume the appearance of one that is carcinomatous, and be easily mistaken for it. The different stages of development of tumors are a further cause of the great confusion which prevails throughout this subject.

The causes of these difficulties are set forth by Müller. Many innocent growths, he remarks, have the peculiarity of progressing in their development with greater or less rapidity. If inflammation should be set up in their substance, solution of continuity may take place in the parts covering them, and a fungoid growth with an ulcerated surface may be formed. The more independent, however, that a growth has become, the further is it removed from the healing, restorative influence of the organism. Moreover, a tumor originally not malignant may, owing to the decomposition which goes on in its inflamed and diseased interior, as well as on its ulcerated surface, exert an injurious influence on the whole constitution; it may give rise to repeated hæmorrhages from its surface, and may induce a state of general cachexy. Should, however, the nidus of these morbid changes be extirpated, their cause is removed; the health will, under favourable circumstances, be restored; and the tumor will not return, unless there exist in the constitution a tendency to the local deposition and organization of the substance which formed the growth. The tumor of aneurism by anastomosis affords an instance of this, as do albuminous sarcoma, and the tumor fibrosus s. desmoides.

The difficulty of diagnosis is greatly increased by the analogous appearance which the malignant and non-malignant tumors often assume when once they have passed into the open state, and continue to grow with an ulcerated and decomposed surface. Thus, aneurism by anastomosis becomes, when in the open state, very similar to fungus hæmatodes in the same condition.

Usually it is regarded as an infallible sign of malignity if a tumor, after having been extirpated once or twice, returns at the same spot, as is so often observed in the case of carcinomatous tumors; but even on this point it is possible to be deceived. The tumor may return because it is not completely extirpated; any part that is left, such is their independence of the general organism, furnishing the nucleus of a future growth.

The diagnosis of morbid growths, Müller goes on to remark, is rendered still more difficult, by the circumstance that, though carcinoma is generally a constitutional disease, yet, under certain circumstances, non-carcinomatous growths may be so too, without necessarily assuming the nature of carcinoma. Tumors formed of tubercular matter, imply the previous existence of the tubercular diathesis. If such a growth is extirpated, it may not return either in its old seat, or in any other part; but, under certain conditions, it may re-appear. This would depend entirely, upon whether the tubercular diathesis still existed at the time when the operation was performed, or whether, though previously present, it had already ceased. Other non-carcinomatous tumors afford instances of similar occurrences; thus, enchondroma of the osseous system may exist in many different parts, in consequence of a diathesis to its formation. The tumors once formed, continue to exist; but if the hand of a patient is removed in after-life, in consequence of the large size which the tumor there may have acquired, the disease will not return in that situation, though it remains unaltered in other parts of the osseous system previously affected during youth.

External, or superficial structural characters being thus insufficient, an accurate microscopical and chemical examination has become a desideratum —in order that we may arrive at all the information which our present methods of investigation bring within our reach. The object of a microscopico-chemical analysis of tumors would be to ascertain whether there are any important internal differences in their organization and chemical composition, and, if they do exist, exactly to determine their nature: it is evident, however, that in practice no such subtle differences can serve as a foundation for the diagnosis of morbid growths. Some pathognomonic characters must, therefore, be found out, such as are easily recognizable, and which do not imply the possession of any peculiar talent, nor of physiological skill. Microscopical and chemical analyses can never become a means of surgical diagnosis. But, if by these analyses we can make out characteristic structural differences in tumors, we *may*, with such internal differences, connect external and easily appreciable ones. A consummation, we apprehend, more devoutly to be wished for than confidently to be expected.

"The means of which we have hitherto been possessed have sufficed, indeed, to distinguish fatty tumors, polypi of mucous membranes, neuroma, aneurism by anastomosis, tumor fibrosus, and some forms of carcinomatous disease, as cancer alveolaris, melanodes, and medullaris. The diagnosis of carcinoma of the mammary gland is still exceedingly difficult, if not impossible; cancer medullaris, too, cannot always be certainly distinguished. Tumors not of a carcinomatous nature have often been confounded with it, because they returned after having been completely extirpated; and, the operation being at length successful, persons have fancied that they had thus cured medullary carcinoma. Scarcely any definite meaning can be attached to the terms sarcoma, steatoma, osteosarcoma, osteo-steatoma, although they are words in current use in medicine, and even in pathological anatomy. A glance at the literature of spina ventosa shews that this obscure appellation has been given to a variety of different diseases of bone; and, lastly, many important forms of morbid structure have been totally undescribed. It must, indeed, be owned, that the greater attention which has of late been paid to the external and internal characters of morbid structure has much advanced the progress of pathological anatomy. Thus, though Mr. Abernethy's attempt at a systematic arrangement of tumors was not very successful, yet his observations, together with those of Burns and Hey, have made us acquainted with the peculiar nature of medullary sarcoma. Equally valuable is the knowledge of cancer alveolaris, for which we are indebted to Laennec, Otto, and Cruveilhier. Fibrous tumors, which certainly are most easily recognized, owing to the peculiarity of their texture, which is usually firm and fibrous, often tendinous and glistening like satin, have been separated from the debatable classes of steatoma and sarcoma, while Bayle has examined their nature with great success. Sir A. Cooper has taught us to distinguish many morbid growths of the breast and testicle; and, further, to him we owe a valuable work on osseous tumors. The various forms of encysted tumor have been well and carefully examined by Dr. Hodgkin. Lastly, we thankfully acknowledge our obligations to the labors of Wardrop, Langstaff, Travers, v. Walther, &c. on carcinomatous growths, as well as to the systematic treatises of Laennec, Cruveilhier, Heusinger, and others." 6.

The upshot, however, of all that has been done or has been attempted, does not, in M. Müller's opinion come to very much, for he asserts that the system followed by most writers is scarcely superior to that adopted by Marcus Aurelius Severinus, whose observations, for the time in which he lived, are excellent.

M. Müller sets forth the time he has expended and the pains he has taken in this investigation, pains and time without which nothing could be done. He arrived at length at the conviction, that in all morbid growths there are constant differences which may be recognized with certainty. The author found that he had formed the most correct notions with regard to those forms which present analogies to healthy structure, as the tumor fibrosus s. desmoides, the albumino-fibrous tumor, enchondroma,—the parallel to cartilaginous structure,—and cellular sarcoma, a parallel structure to the tissues of the chorda dorsalis, and of the decidua, which are composed of cells analogous to those of plants.

"As early," he proceeds to say, "as the year 1836, the author had recognised with the microscope the cellular structure of various morbid growths; namely, of laminated cholesteatoma, of cellular polypi, and of osteo-sarcoma. At the same time the author pointed out the analogy between cholesteatoma with its polyedrous cells, resembling those of vegetable parenchyma, and that cellular structure of the chorda dorsalis which he had been the first to demonstrate. Enchondroma also was described as a structure parallel to cartilage, both in anatomical and chemical characters, and sufficiently distinguished, by the presence of chondrine, from other morbid growths. Fibrous structure was shewn to predominate in the tendino-fibrous and albumino-fibrous tumors, as well as in carcinoma hyalinum. Very regular crystals were observed in many growths, and several new and peculiar forms of carcinomatous degeneration were described. The presence of caudate bodies, as a primary element of morbid structure, was soon afterwards ascertained by the author, who gave a description of them as they exist in fungus medullaris, and in melanosis." 8.

Nuclei, cells, caudate bodies, fibres, and crystals, are the only elementary bodies which have hitherto been discovered. Cells would seem, from the researches of Schleiden and Schwann to be of great importance, all the tissues of the embryo, according to the latter, being formed from cells, which are themselves developed from nuclei; growth being the result of fresh formations of cells, which afterwards undergo transformation into other tissues. M. Müller set himself to work to test the application of Schwann's observations on healthy to morbid structures, and he found them correct; for, by employing a high magnifying power, cells were observed in many morbid growths, in which they were not previously known to exist, as in collonema, in many varieties of carcinoma, and in enchondroma. In most growths presenting a cellular structure, with the exception of cholesteatoma and cellular polypi, the nuclei of the cells were discovered, situated either in their walls or in their interior: in many instances, too, young cells were formed within older ones, as was the case in sarcoma, enchondroma, carcinoma, and collonema.

### 2. *Chemical Examination of Morbid Growths.*

As morbid structures present elementary forms common to the normal, so their chemical constituents offer analogous resemblances. It is perhaps by the conjunction of the slight differences of either kind, that peculiar characters can alone be made out.

Chemistry points out three grand differences between morbid growths. The proximate animal principles which exist in them are composed either of some kind of fat or gelatine, or of albumen. This, of course, is not asserted strictly, but only with reference to the mass, since other materials, as osmazome, salivary matter, caseine, &c. are present in small quantities. The division of morbid structures into the three classes distinguished by the presence of fatty matter, of gelatine, and of albumen, is moreover only relative, for those materials occur combined in pathological products. It is therefore only by the predominance of one of the three ingredients in question, that the leading chemical character of a morbid growth will be determined. The classification is a loose and general one.

*a.* "*Fatty Tumours* present many varieties, partly in their structure, partly in the nature of the fat they contain. They always consist of an animal, organized base, composed, not of fat, but of cells or cysts secreting it, and of the fat itself. Their nature may be, in part, determined by the physical appearance of the fat: in part it is discovered by the action of certain re-agents; some fat being capable of conversion into soap, while another kind of fat is incapable of undergoing that process. The fatty constituent of these growths is fusible at a certain temperature, greases blotting paper, is extracted by hot alcohol or ether, and is again deposited in forms either crystalline, or irregular, on cooling or evaporation. To this class, lipoma, or the common fatty tumor, may be referred, and cholesteatoma, or the laminated fatty tumor." 11.

In the structures of which fat is not the chief element, it generally exists either in the form of oil-globules, or of granules, or of minute crystals.

*b. Gelatinous Morbid Growths.*—These are recognised by their almost entire reduction by long boiling to gelatine. The length of time requisite for this differs in different cases. In many instances from ten to twelve hours suffice; eighteen or more hours are often requisite; and to effect a complete solution of all parts containing gelatine, a longer period is frequently necessary. Growths yielding gelatine, when once known, may easily be recognized. The gelatine obtained by boiling, whether chondrine or ordinary gelatine,—*colla,*—is naturally mixed with osmazome, which must be removed. Among the class of growths yielding gelatine may be ranged the cellulo-fibrous tumor, the tumor fibrosus s. desmoides, enchondroma, and the osteoid tumor.

*c. Albuminous Morbid Growths.*—" These either yield no gelatine, even when boiled from eighteen to twenty-four hours; or if, after long boiling, some gelatine should appear, still by far the greatest portion of the growth remains undissolved, thus shewing, that some substance of an albuminous nature is its main constituent. The water in which these structures have been boiled may contain, even after having been filtered, a substance soluble both in hot and cold water. Some of these growths yield a trace of caseine, which may be detected, in addition to the ordinary not very decisive tests, by a little acetic or hydrochloric acid, which renders the filtered fluid turbid, but restores its pellucidity when added in excess. The fluid is also rendered turbid by alum, but does not recover its transparency on the addition of an excess of that substance. Caseine exists in a very minute quantity in most albuminous structures, as in albuminous sarcoma, in carcinoma simplex, or reticulare. Sometimes, also, these growths contain a substance closely allied to salivary matter, and precipitated neither by acids, alkalies, metals, nor earthy salts, and even not by alcohol nor tannin. The presence of this matter can be discovered only negatively, by evaporation to dryness and carbonization. The author found it in collonema, and in carcinoma alveolare; in the gelatiniform mass of which latter growth no caseine could be detected." 13.

When not kept long in spirit, these growths contained osmazome.

The external appearance of albuminous structures is sufficiently striking. They never have a compact, tendinous texture; sometimes they are tender and gelatiniform, like the chorda dorsalis; at other times fibrinous, like decidua; sometimes they are cellular, granular, or fibrous; generally they are friable and easily torn. To this class belong collonema, scrofulous tumors, albuminous sarcoma, albuminous osteo-sarcoma, and all varieties of real carcinoma. If gelatine be present, it is probably from the cellular tissue in the growths.

### 3. *Microscopic Characters of Morbid Growths.*

The minute microscopic elements of morbid growths are, in addition to capillary vessels; fibres, granules, cells both with and without nuclei, caudate or spindle-shaped bodies, and vessels. Our author has detected blood-vessels in all morbid growths except cholesteatoma, which is often developed within cysts, with which it maintains no organic connexion. M. Müller scouts the idea of scirrhus being unorganized; and he also repudiates the notion of the arrangement of the vessels in any tumor, except in aneurisms by anastomosis, presenting any thing remarkable. Injections, therefore, have done little service in this direction, and they have the disadvantage of concealing important forms of structure, such as may be satisfactorily studied by means of the microscope, in fresh specimens, or even in such as have been kept for some time in alcohol.

*Fibres* are a grand constituent both of the albuminous and gelatinous growths. Thus, the cellulo-fibrous tumor, and the tendino-fibrous tumor, are composed of fibres; and so are the albumino-fibrous tumor, and the carcinoma fasciculatum, seu hyalinum. In the cellular albuminous tumor, fibres are quite a secondary element. In carcinoma alveolare, the walls of the old cells, in which the generation of new cells takes place, become at last split into isolated fibres, having scarcely any connexion with each other. Occasionally, too, the caudate cells, when arranged in a certain manner, produce in albuminous growths an approximation to fibrous structure.

*Granule* is the name applied to such spheroidal or elliptical bodies as do not present any internal cavity when viewed under the microscope. They are present in vast numbers in some albuminous tumors. Often, too, in different specimens of carcinoma, the germinal cells were found to contain, in addition to the formative globules and young cellules, a number of small granules perfectly distinct from both. In carcinoma fasciculatum, many round granules are situated between the fibres.

The *Cell* is by far the most frequent element of morbid growths. Thus, it exists in sarcoma cellulare, in enchondroma, in carcinoma simplex, reticulare, and alveolare. In many growths this cellular texture is so coarse as to be evident by a very low magnifying power, or even to be distinguishable by the naked eye; but, generally, the cells, unless magnified 400 to 500 times, look like granules.

"Cells sometimes form the only tissue of morbid growths, as in the case of cholesteatoma, of carcinoma alveolare, of cellular sarcoma, and of osteo-sarcoma. In these instances, cells cohering by their walls form the important parts of the structure, while fibres of cellular tissue serve only to form membranes uniting together its several lobuli.

In other cases, where microscopic cells still form the basis of the growth, these cells do not cohere, though in close apposition; but, on the contrary, are free, may be separated from each other, and, when looked at under the microscope, present the appearance of globules. It is only by employing high magnifying powers that their real structure becomes evident: then they are seen to be spheroidal cells, the presence of a small cellule, or of several corpuscules in their interior, serving to mark their cavity. These minute globular cells, which form the real *seminium morbi* in several forms of carcinoma, as in carcinoma simplex, reticulare, and alveolare, are deposited in extraordinary number in the meshes of a fibrous texture." 16.

*Nuclei.*—The presence or absence of a nucleus in the walls of the cell constitutes a structural difference between morbid growths. The nucleus, when present, is situated in the substance of the wall of the cell, and from it the cell has been developed. Sometimes also a cell contains within its cavity nuclei, which serve as the germs of new cellules. This is the case in enchondroma, and in carcinoma alveolare. In most cases, as in enchondroma, in several forms of carcinoma, in cellular sarcoma, and in osteo-sarcoma, the parietal nucleus, at least, of each cell is evident, and may be recognized by its flat or roundish form, and generally by its darker tint. In other instances, however, the cells have no nuclei; of this, cholesteatoma affords an example.

Some normal cellular structures, cartilage for instance, yield gelatine—so does the cellular pathological structure, enchondroma. The cellular textures, the chorda dorsalis, and decidua, yield no gelatine—nor do the parallel pathological products—gelatiniform tumors and cellular sarcoma.

*Young Cellules.*—"Some cells do not contain *young cellules*, while within others a series of younger ones are encased one within another, and this furnishes us with a new means for distinguishing between different morbid growths. Laminated cholesteatoma affords an example of the former kind, for it consists of a polyedrous cellular tissue, resembling vegetable parenchyma, in which the author has never detected smaller cellules. In other cases the cells are encased, one within the other. A cell, when viewed under the microscope, appears to contain corpuscules in its interior; but careful examination shews, in accordance with Schwann's discoveries as to the primitive formation of healthy tissues, that these corpuscules are either young cells, one contained within the other; or nuclei, from which young cellules are developed. This holds good of many of the cells in sarcoma cellulare, in carcinoma alveolare, and in enchondroma, as also of some formative globules in carcinoma simplex and reticulare. The finest cells can be recognised only by the most powerful magnifying-glasses, and often are not larger than 0.00015 to 0.00021 of an English inch: the average size of the cells in growths with a cellular base is 0.00054 of an English inch." 17.

*Caudate Bodies.*—Another frequent element of morbid growths. They are elliptical pouches, or cells, terminating at one or both extremities in a fine caudiform fibril of uncertain length. Sometimes the interior of these bodies is granulated, and filled with a greater or less number of granules. The interior of their cavities is, however, seldom distinctly visible, though occasionally a nucleus, of a somewhat darker tint than the surrounding substance, may be observed, together with one or more nucleoli. Schwann observed the same structure in primitive cellular and in other tissues undergoing the transformation from cellular to fibrous structure. Elongated cells become fibres, and thus do most fibres in the animal body seem to be formed: but in those structures which consist of caudate corpuscules, it would appear as though the development of the fibres had been arrested while they were in the half cellular state in which they exist in the embryo. Frequently, one end only of the corpuscules is prolonged into a fibril, while the other remains obtuse. There is a great difference, too, in the length of the fibril: sometimes it is no longer than the corpuscule, or even does not equal it in length, while at other times it greatly exceeds it. The diameter of each fibril is generally from $\frac{1}{2}$ to $\frac{1}{7}$ only of that of the corpuscule. In no case has the author observed several corpuscules connected with one fibril. In some instances, however, not only does each end of the corpuscule give origin to a fibril, but a third springs from its side, and sometimes the fibril which proceeds from the end of the corpuscule becomes bifurcated at its extremity.

The arrangement of the caudate corpuscules often presents great varieties, sometimes a few only being found in fungus medullaris, at others there being so many as to constitute the main bulk of the tumor.

The caudate corpuscules are by no means peculiar to fungus medullaris; they may, indeed, often be observed in its substance, but often they do not exist in it, while they are as frequently met with in non-carcinomatous as in medullary growths. They occur in the substance of aneurism by anastomosis, and in one instance the author saw them in an albuminous osteo-sarcoma of the lower jaw, which was extirpated with success. On another occasion a large benignant fungus of the conjunctiva, of the nature of albuminous sarcoma, was made up almost entirely of these corpuscules. In no instance has the author met with them in large number, in those tumors which become converted into gelatine by boiling. Nevertheless, it is possible that, even in these latter growths, they may be found at certain periods in considerable number, for they probably depend only on the transformation of cells into fibres, and consequently are merely fibres in an early stage of development.

### 4. *Development of Morbid Growths.*

"Differences in the structure of the microscopic forms of morbid growths depend on the way in which their development proceeds. Cellular growths are those in which the process of development can be most easily traced, now that Schwann has laid the foundation for such investigations by his discoveries with regard to the development of healthy tisues. According to Schwann's observations, nearly all animal tissues are, in their primitive form, composed of cells, which have precisely the same structure as those of vegetables, and their formation and growth are regulated by laws exactly resembling those to which Schleiden discovered the cells of plants to be subject. In the wall of each young cellule is a nucleus from which it is developed. New cells are formed either within the interior or on the surface of old cells: in the former case they are developed from a nucleus loose within the cavity of the parent cell, and unconnected with its parietes. Schwann has demonstrated the former process as it occurs in cartilage, and in the chorda dorsalis: the latter appears to take place in the case of many other textures, for Schwann has shewn that all tissues in the embryo consist of cells with parietal nuclei, though it is not possible in all to prove the formation of new cells in the interior of old ones. An instance of the constant formation of new cells with parietal nuclei, external to the old cells, is afforded in the adult by the cells of the epithelium, which do not display any approach to an endogenous mode of growth. The formation of young cells can, however, be best observed in those cases in which they are developed internal to the old ones, or, in other words, in which the nuclei of new cells are situated within the cavity of old cells. In these cases the process of development as observed by Schleiden in vegetable tissues, and by Schwann in those of animals, is as follows:—

The nuclei protrude young cellules, which project from their surface as the watch-glass from the watch. As growth proceeds, the young cell increases in size, while the nucleus remains imbedded in its wall. If several young cells should be formed from several nuclei seated within the parent cell, they progress in growth so as to fill up its cavity, and then their walls usually become confounded with those of the parent cell. Fresh nuclei form within the cavity of these young cells, and from a repetition of this process result successive generations of cells. The walls of the young cells are perfectly transparent, but those of the older cells become thickened, and, in animal tissues, often converted more or less into a fibrous structure. In this way the cells of cartilage and of the chorda dorsalis, probably also those of the decidua, become developed." 32.

It would be natural, observes our author, to expect a similar process in the formation of many pathological structures. The young cells in enchondroma and in cancer alveolaris are formed in a precisely similar way; and the author's observations render it probable that the same process also obtains in the development of many forms of carcinoma, and of cellular sarcoma. Albumino-cellular sarcoma and osteo-sarcoma, and gelatiniform sarcoma, appear to be developed in this way, as is also collonema. In many of the parent cells the young cellules with their parietal nuclei, were very distinctly seen. Successive series of cells, encased the one within the other, are seen to make up the structure of carcinoma alveolare. The large cells, which are visible to the naked eye, contain in their cavities a second generation and so forth, till we arrive at the smallest cells of all, in which are nuclei of a darkish yellow tint, generally somewhat elongated, and presenting a minutely granular structure. Here and there these nuclei may be seen lying free in the cavity of the cells; in other parts they have already evolved a germinal cell, and may be seen imbedded in the substance of its parietes. The walls of the large cells, when greatly developed, appear to assume a fibrous structure, and at last to burst.

The formative globules of simple and of reticular cancer of the breast are not merely cells containing granules, but sometimes in the interior of each are one or more roundish or elongated germinal cells, with a darkish nucleus in their parietes, from which they have probably been developed.

How similar soever, adds our author, the most different morbid growths may be at their first origin, still they present great diversities in their subsequent development—a fact which he illustrates by what takes place in carcinoma alveolare, and in carcinoma simplex and reticulare.

5. *Development of Caudate Corpuscules.*

"It results from Schwann's observations on the tissues of the embryo, that these bodies are cells which have undergone a metamorphosis. In proof of this may be adduced the fact, that not only has the author seen caudate corpuscules in carcinoma medullare scattered among cells; but likewise in a specimen of sarcoma with causate corpuscules in the midst of the fasciculi of these bodies were cells, some elongated, and others round, containing a germinal cellule with its parietal nucleus. In this instance the greater part of the morbid growth presented a fibrous appearance, owing to its being formed of caudate corpuscules arranged in fasciculi; but towards the surface the fibrous part seemed to assume a granular structure, and examination with the microscope shewed that there cellular globules existed in place of the caudate corpuscules. In melanosis some of the cells containing pigment were seen by the author to be round or oval, while others had a caudate foam. Lastly, caudate corpuscules are sometimes seen in which there is a distinct cavity. The external skin of the fœtus is, according to Schwann's observations, entirely formed of caudate corpuscules which terminate in long fibres, and constitute the fibres of the skin. In many other situations, too, Schwann saw caudate corpuscules in cellular tissue. These facts satisfactorily account for the presence of caudate corpuscules in innocent as well as in malignant growths. They are, like the germinal cells, an embryonic formation; and embryonic formations are found to be repeated in a remarkable manner in morbid growths." 24.

The consequence of these observations would seem to be highly important—for they upset the division of pathological structures into homologous and heterologous—a division, says M. Müller, founded on "gratuitous hypothesis." Some other pathognomonic characters of carcinomatous growths must be sought. The distinction between the carcinomatous and the innocent forms of albuminous growths presents the greatest difficulties. Here, neither the minute structure nor the chemical characters of the growth can be our guide, for carcinomatous tumors belong to that class the main constituent of which is a substance yielding albumen; consequently there are malignant albuminous growths. On the other hand, the diagnosis between carcinomatous structures and such as yield gelatine is very simple.

"Whether the carcinomatous diathesis be peculiar and distinct from all others, or whether, under certain circumstances, any other structure may pass into the state of carcinoma, still the same question presents itself;—is there any other characteristic of carcinomatous growths than such as are derived from their minute structure, or from the process of their development? The solution of this question must always be the grand problem in the anatomy of morbid growths. The examination of numerous specimens of carcinoma has taught the author that they are, indeed, possessed of certain peculiar anatomical characters, which may serve to identify them ; and, further, that these characters are distinguishable, on making a section of the growth, either by the naked eye, or at any rate by the aid of a common magnifying glass.

Although the structures which belong to this class are extremely various, still one may take the place of another. After the extirpation of carcinoma simplex, for example, carcinoma alveolare or fasciculatum may succeed, and often the several forms are coexistent. But, although transitions of the different forms into each other may occur, yet their extremes are very dissimilar, and no sort of resemblance can be traced between carcinoma simplex, or scirrhus, and carcinoma fasciculatum. In order, therefore, to be able to recognise carcinoma, a person must make himself acquainted with the individual peculiarities of every form of morbid structure, both innocent and malignant: in short, he must proceed as the botanist does who busies himself with the study of poisonous plants." 26.

M. Müller is convinced that a tolerable degree of accuracy and certainty may be attained, in diagnosis, but he admits that there are some forms of disease, which, being destitute of any well-marked external peculiarities, may be readily confounded with others.

The principles, he winds up this introductory chapter by remarking, in accordance with which morbid structures must be classified, cannot be exclusively derived either from their minute structure, or from their chemical composition. For growths widely differing in their physiological characters and in their susceptibility of cure may present a perfect indentity in their minute structure: similarity of structure may coexist with differences in their chemical constituents, or the same chemical characters, may be found in growths, between which the greatest diversity exists with regard to their structure, physiological characters or curableness. In determining the different genera, therefore, the subject must be regarded in all these points of view.

M. Müller passes to the particular consideration of the several morbid growths. He first treats of the minute structure of carcinomatous growths —and then examines those morbid growths which may be confounded with carcinoma. The present publication is only the first Part of the entire work; the second is to appear hereafter. The importance of the subject, and the commensurate importance of the manner in which it is handled, furnish ample reasons for our noticing it in the fullest manner,

### On the Minute Structure of Carcinomatous Growths.

M. Müller opens this section with some observations on carcinomatous growths in general—and then takes up successively—scirrhus, or carcinoma simplex—carcinoma reticulare—carcinoma alveolare—carcinoma melanodes —carcinoma medullare—carcinoma fasciculatum, and winds up by an account of the development and softening of carcinoma—the chemical properties of carcinoma—and the nature of it. To each of these subjects we shall now advert.

### 1. *On Carcinomatous Growths in General.*

M. Müller defines those growths as cancerous, which destroy the natural structure of all tissues, which are constitutional from their very commencement, or become so in the natural process of their development, and which, when once they have infected the constitution, if extirpated, invariably return, and conduct the persons who are affected by them to inevitable destruction.

It appears to us that such a definition is unsatisfactory, and possibly not altogether correct in a practical sense. It is unsatisfactory because it rests on a character difficult to be determined—the implication of the constitution; and it may be incorrect, because we cannot say that, even if the constitution be involved, the tumor must inevitably return after extirpation, and destroy the patient. No doubt it will do so in the immense majority of cases, but we know too little of the amount and the nature of constitutional implication, to pronounce so authoritatively on its effects. We freely admit, however, that the definition is as good an one as can be given, and if it leans on the side of the malignancy of cancer, so much the better for surgery and for humanity, so much the better for the repression of those cruel and useless operations which *were*, at all events, too frequently resorted to.

The forms of disease, observes our author, which may be classed under this head are extremely various, though in some cases they pass into each other by imperceptible gradations. This fact and the circumstance that, after extirpation of the disease, one form may take the place of another, serve to exhibit the physiological connexion between growths, the extremes of which often do not shew even the most remote similarity of structure. The different kinds of carcinoma may either succeed each other, or may co-exist.

The most invariable anatomical character of the carcinomatous degeneration is loss of the proper tissue of the affected part, which always disappears during the progress of cancer. However dissimilar the tissues, all become involved in the same cancerous degeneration.

It is, however, in the interstices of healthy tissues that the elementary forms of carcinoma are at first developed. In these interstices are found the germinal cells of cancer, a real *seminium morbi.* This is seen excellently well, when the muscular coat of the stomach is affected with carcinoma alveolare. The germinal cells of carcinoma are deposited between the bundles of muscular fibre, which in the early stages of the disease are easily distinguishable: at even a later period the muscular layer of the stomach, though enormously swollen, may still be recognised, until at length the production of the germinal cells equally in all the coats of the stomach obliterates every trace of their different layers, and of the natural structure of the organ.

The parts in the neighbourhood of a cancer usually become firmly connected with it at an early period, hence carcinoma is less moveable than other growths. In the female breast, this adhesion to the pectoral muscles and skin, as well as the retraction of the nipple, are remarkable—both, however, *may* be absent. M. Müller dilates on the anatomical characters of carcinomatous disease of the walls of the stomach, but it does not seem necessary for us to follow him.

He observes that eccentric development is not peculiar to carcinoma, nor does softening always begin at the centre of the growth; nor is it always characterized in its early stages either by lack of vessels, or by any peculiar distribution of them. The vessels in it bear the same relations as in other parts: sometimes they are scanty, at other times exceedingly numerous.

He adds:—"The positive characters of carcinoma do not display any thing heterologous or foreign to healthy organization: some of the elements of cancer occur in the healthy organism of the adult, while others are such as exist in the primitive fœtal state of tissues, as cells, varicose fibres, and cylindrical fibres. Varicose fibres are produced by the elongation of cells and their linear arrangement, and perfect fibres are, in their turn, formed from such as are in the varicose state; whence it follows that the differences of the extremes depend merely on the point at which the development of the tissues is arrested. A structure, the development of which is arrested while the cells are in their primary state, will be very unlike one in which the cells are elongated, and in progress of transformation into fibres; while those growths which tend rapidly to assume a fibrous texture will also present a different appearance.

An albuminous substance forms the basis of all carcinomatous growths; for, if freed from skin and cellular tissue, they may be boiled for eighteen or twenty-four hours, without yielding more than a very small quantity of gelatine; often, indeed, without the slightest trace of it being discovered. The author has often repeated this experiment with carcinomatous growths, and always has arrived at the same result, namely, that the mass of carcinoma is perfectly insoluble in water. What little of it is dissolved occasionally contains caseine and salivary matter." 32.

We think that these observations will put an end to the confident anticipations expressed by some pathologists, and entertained by many persons in the profession, that some simple microscopic test of malignant growths would be discovered. Our readers may remember, that, in our notice of Dr. Hake's paper upon "Varicose Capillaries," we gave expression to our doubts of the importance of the observation, remarking that it was consistent with reason to suppose that in morbid growths, whether malignant or otherwise, the capillary vessels would be likely to enlarge and become varicose. The consistence of such doubts with the results of extended investigation will be evident from what M. Müller has stated. An instance this of the caution requisite in giving public expression to philosophical discovery.

Having made these observations on carcinomatous growths in general, M. Müller passes to

*Scirrhus, or Carcinoma Simplex, or Carcinoma Fibrosum.*

M. Müller observes that, prior to the discovery of medullary sarcoma by Mr. Burns, and of cancer alveolaris by M. Laennec, this was regarded as the only form of cancerous degeneration. Most descriptions, therefore, of cancer of the breast, by the earlier writers, refer to this variety of morbid growth, characterised by its almost cartilaginous hardness, by its being irregular in outline, seldom lobulated, presenting a grey appearance when divided, and generally giving rise to connexions between it and the skin, and to retraction of the nipple.

M. Müller cites the observations and descriptions of Adams, Baillie, and a host of others, but, passing over these, we shall content ourselves with bringing forward his own account of the appearance of common scirrhus of the mammary gland.

"The diseased masses are generally irregular in form, not lobulated, hard, and resisting the knife, and presenting, when divided, a greyish appearance, which has but very little similarity to cartilage. Whitish bands are not invariably present. Scirrhus of the mammary gland occasionally shews, here and there, whitish filaments some of which are hollow, and contain a colourless, whitish, or yellowish matter. Probably this appearance of white filaments is the result of thickening of the walls of the lactiferous tubes and lymphatics, and this idea is confirmed by the absence of these filaments from scirrhus of non-glandular parts. The mass of scirrhus is composed of two substances, the one fibrous, the other grey and granular. The fibrous substance is rarely apparent immediately on making a section of these growths, but is seen on scraping away the grey matter, for which it serves as a sort of basis. On removing the grey matter, either by scraping it away or by maceration, the fibrous substratum is seen to be composed of a very irregular network of firm bundles of fibres. The grey matter is found to consist of microscopic, formative globules, but slightly adherent to each other. These globules may be seen on examining fine sections of scirrhus with the compound microscope, or, still better, by scraping out the grey matter and examining it alone. The formative globules are then seen to be transparent, hollow cellules, from 0.00048 to 0.00108 or 0.00130 of an English inch in diameter. They are insoluble in acetic acid, and also in water, at any temperature. In many of these cells, only a few points, which look like small granules, can be seen; while in others a larger body may be distinguished, which looks like a nucleus, or like a smaller vesicle, contained within a formative globule. In many scirrhous breasts which the author examined, he was unable to convince himself of the presence of smaller cellules within the formative globules, while in other instances their existence was distinctly recognized. The appearance of these smaller vesicles within the larger seems to depend on the formative globules being in the stage of development. In one case of exceedingly hard scirrhus of the breast, which had passed into the open state, many formative globules were seen in the condition represented at Plate ii, fig. 14*. In several of these cells no vesicular content was observed, but in many others, under a high magnifying power (from 400 to 500 times), one or two smaller cellules were seen, each of which was furnished with a small, darkish corpuscule—a nucleus. Though crowded closely together, the formative globules lie between the meshes of a fibrous structure, with which they have no connexion, and from which they can be easily removed, while, notwithstanding the thinness of their walls, they can be isolated from each other with the greatest facility." 43.

M. Müller goes on to say that it is difficult to make out whether the single or double vesicular corpuscule, which is often distinctly seen within the formative globule, corresponds to the nucleus of a cell, or whether it is a young cell encased within the old one. He inclines, however, to the opinion that the vesicular bodies do correspond to young cells.

Since, he remarks, many structures in the embryo are originally developed from cells, there exists a general resemblance between the cellular texture of carcinoma and the primitive state of those tissues. But the analogy is only general, carcinoma not resembling one tissue more than another. In addition to the formative globules of carcinoma, oil globules are numerously diffused through scirrhous growths.

---

* "The author gave a short notice of this structure in a postscript to an article by Schwann, in Froriep's Notizen, 1838. Januar. No. 3."

*Carcinoma Reticulare.*

M. Müller believes that he was the first to describe this form, a more frequent one than even carcinoma simplex in the female breast. On making a section of it, he says, it may be immediately distinguished from the latter by the white reticulated figures intersecting the grey mass, which are perfectly evident to the naked eye. It acquires a large size more readily than carcinoma simplex, and is further distinguished from it by its tendency to assume a lobulated form. It sometimes approaches the consistence of scirrhus; at other times it is softer, and more nearly resembles fungus medullaris. A great number of observations have convinced the author that the consistence of this form of carcinoma is very variable, while its structure always remains the same, and is so peculiar that it may in all cases be recognised by the naked eye on making a section of the growth. Indeed, with the exception of cancer alveolaris, no form of carcinoma can be so readily distinguished. By far the greater number of cancerous degenerations of the female breast belong to this class, and more than thirty recent specimens of it have come under the author's notice within the last four years.

Carcinoma reticulare is not unfrequent in other organs. M. Müller saw it in the swollen axillary glands in a case of cancer reticularis of the mamma: once he observed it in the stomach, and he has seen it both in adults and in children in tumors of the orbit and of the ball of the eye, attended with complete degeneration of the muscles of the eye, of its coats, and of the optic nerve. He has only once found it in cancer of the lip, and once, in enormous masses, in the anterior mediastinum, similar small growths having formed on the surface of the heart.

"Carcinoma reticulare is composed of a grey mass made up of globules, and imbedded in a reticulated fibrous tissue, which is not seen until after the removal of the grey granular mass. The grey mass consists of transparent formative globules or cells similar to those of carcinoma simplex. These globules likewise often contain two or more smaller vesicles with nuclei of a pale colour. In other cases, however, the smaller germinal cellules could not be distinguished within the interior of the larger formative globules, which were then found to contain a number of small granules. Occasionally these granules were present in great number in the interspaces between the cells, and in some of them a molecular motion was distinctly evident. The cells themselves had a diameter of 0.00022 to 0.00039 or 0.00043 of an English inch, while the diameter of the granules contained within them did not amount to one-fourth or one-fifth of that size.

The white, or yellowish-white reticulated figures which are always more or less distinctly evident in this form of cancer, present a very peculiar appearance. These figures are irregularly reticulated: sometimes they present a branched arrangement, at other times they appear in spots. They are peculiar formations, not dilated vessels with thickened parietes, such as are sometimes seen in carcinoma simplex, but they are produced by the deposition of grains of white matter in the grey mass. These grains do not appear to be cells, but generally seem to be made up of opaque granules agglomerated together, so as to form roundish or elongated corpuscules. These corpuscules are usually of a round or oval shape; sometimes, however, they are elongated, and it is not uncommon for them to be much longer than they are broad. They are two or three times as large as the red particles of the blood, their greatest diameter being 0.00076 of an English inch. This structure of the white substance of carcinoma reticulare is not, however, in general evident to the naked eye, nor even with the aid of a lens ; but the corpuscules of which it is composed are usually so distributed through the grey matter, as to present the appearance of a white net-work.

If these white figures are examined under the simple microscope with a magnifying power of eight or sixteen diameters, the corpuscules of which they are composed may be distinguished.

A higher magnifying power exhibits the granular appearance of these corpuscules, which, when seen by transmitted light, of course appear dark. Lastly, we may isolate these bodies under the compound microscope, and convince ourselves that they are formed by the agglomeration of small granules, either perfectly opaque, or but slightly pellucid. These white granules are not rendered transparent either by acetic acid or by alcohol." 47.

The white corpuscules accumulate with the development of the disease, and form, by the time that disorganization has commenced, a large part of its structure. They constitute a portion of the softened matter and purulent secretion yielded by the ulcerated surface.

Occasionally, in carcinoma reticulare of the female breast, cavities form in the interior of the structure. Once, the author observed a large cavity, the walls of which were completely occupied by white corpuscules. Masses of this sort extend, still preserving their reticulated figure, and may be separated in large portions of a soft consistence, for the purposes of chemical analysis. The matter composing them is found to be very similar to coagulated albumen. As the disease advances, the reticular figures become confluent, and appear like irregular white spots.

"In one specimen of cancer reticularis mammæ, the author observed throughout its tissue many little cavities, varying in size from that of a millet seed to that of a pea, filled with a yellow, puriform, or cheesy matter. These cells were furnished with distinct walls, but their cavities communicated here and there with each other, so that the contents of several cells could be squeezed out through an opening in one. The larger ramifications of these cells gave off smaller branches. The interior of even the smallest cells, the diameter of which did not exceed half a line, was found to be furnished with a distinct lining membrane, to which blood-vessels were distributed. The matter which these cells contain must not be confounded with the white globules strewn through the reticulated figures, although it is possible that both may have a common origin. In the instance just spoken of, the white reticulated figures formed here and there large masses, which were merely imbedded firmly in the surrounding tissue, and did not exhibit the slightest approach to a cellular structure. In another part of the same morbid growth was a carcinoma alveolare of the size of a hazel nut, the cells of which were filled with a jelly-like matter. The author has frequently seen patches of carcinoma alveolare, forming part of the morbid growth, in cases of carcinoma simplex mammæ." 49.

Carcinoma reticulare is sometimes slowly, sometimes rapidly, developed. In far the greater number of cases it returns after extirpation. But, in one instance, Professor Pockels removed a cancer of this sort with complete success—in another case, the disease has not yet returned, though two years have passed since the extirpation ; this was a case of carcinoma reticulare complicated with carcinoma melanodes of the ball of the eye and orbit, in a young woman—in another case of carcinoma mammæ the disease had been removed in the other breast five years previously.

*Carcinoma Alveolare.*

Described in Germany by Otto, as a peculiar species of scirrhus of the stomach, and, in France, by Laennec and Cruveilhier as "cancer gelatiniforme" and "areolaire." M. Müller takes Otto's account of the appearances in a case, as exactly representing the general anatomical characters of the disease.

The scirrhus occupied more than two-thirds of the whole stomach, and extended from the pylorus over a width of more than seven inches of its anterior and posterior wall. The walls of the stomach were so thickened at the diseased parts, that they did not collapse. In several places they were two inches and a half in thickness. The surface of the scirrhous part was uneven and tuberculated. Otto says that its substance differed so much from that of ordinary scirrhus, that, perhaps, it ought not to be referred at all to that class of diseases. The basis of its structure was composed of innumerable white fibres and laminæ crossing each other in all directions, and having their interspaces occupied by cells which varied in size from that of a grain of sand to that of a very large pea. Some of the cells were closed, but many of them communicated with each other: they all contained a very viscous, clear, perfectly transparent jelly. Externally, the diseased growth was covered by peritoneum, through which the half-projecting sacculi and cells were seen. The inner surface of the stomach was almost entirely deprived of its lining wherever the disease extended, and most of the cells, both large and small, opened into the cavity of the stomach, into which, when firmly pressed, they poured their contents. The inner coats of the stomach were entirely destroyed by the disease, the muscular coat extended for a short distance into the morbid structure, but small cells filled with jelly-like matter were everywhere deposited between the muscular fibres.

M. Müller adds, that, at the commencement of the disease, the mucous and muscular coats of the stomach swell, and sections of the latter present that striated appearance observed in all forms of carcinoma of the stomach. The cellular structure containing the jelly-like matter develops itself between the bundles of muscular fibre, but the like process takes place at the same time in the mucous membrane. In the early stages of the disease the cells can be distinguished only by means of the microscope.

Occasionally, this cellular structure not only becomes developed in the stomach, but also forms isolated nodules in different parts of the surface of the peritoneum.

The symptoms of this form of cancer of the stomach are peculiarly obscure. It is especially slow in producing the cachexia cancerosa, but the peculiar colour of the face characteristic of organic disease of the stomach has not been absent. Cancer alveolaris, however, is not confined to the stomach. Cruveilhier has seen it in the small intestines, in the rectum, cæcum, uterus, ovary, and in the bones; and the author has also met with it in the intestines, in the female breast, in the great omentum, and especially in the peritoneum. The jelly-like matter contained in the cells yields no trace of gelatine.

"Cruveilhier distinguished between 'cancer areolaire gelatiniforme,' and 'cancer areolaire pultacé:' the cells of the former contain a transparent jelly, those of the latter a turbid pultaceous matter. He has observed the latter form in the uterus and in the bones. The case which he relates of 'cancer alveolaire pultacé' of the skull is very remarkable, and the representation he gives of it is very interesting. The diseased bones were the frontal, the ethmoid, the inferior turbinated bones, and the vomer. The morbid growth extended both outwards and inwards, and had attacked the mucous membrane of the nose, and the dura mater." 52.

M. Boutin Limousineau analysed the yellow matter from the cells, and found that it contained caseine. A somewhat similar structure was observed by M. Müller in a breast removed by M. Dieffenbach.

"The author obtained the following results* from microscopic examination of carcinoma alveolare of the stomach. If the smaller cells are looked at under the microscope, they are found to contain encased within them many still smaller cellules, which in their turn include others of yet more diminutive size. In the smaller cells the darkish yellow parietal nucleus is distinctly evident. Many cells, likewise, contain mere nuclei, free within their cavity, as cystoblasts from which new cells are to be afterwards developed. The walls of the largest cells are distinctly fibrous, and their fibres run from one cell to another. Twice the author observed rod-shaped crystals in the jelly-like matter of preparations which had been kept in spirit; and on another occasion he saw spindle-shaped corpuscules in the jelly from a cancer alveolaris of the breast.

The history of the development of carcinoma alveolare corresponds exactly to that of the primitive formation of cartilage and of the chorda dorsalis, as described by Schwann. The young cells are produced from cystoblasts, or nuclei developed in the interior of the parent cell; and although the parent cell continues to increase in size, these young cellules by degrees fill up its cavity. At length the walls of the young cells come in contact with each other, and they form together with the parent cell, within which they are encased, one compound cell. Thus, the process of development goes on, till the larger cells on the internal wall of the stomach burst, and pour their jelly-like contents into its cavity.

The fibres forming the walls of the largest cells constitute a nidus within which younger generations of cells are developed. In order, however, to observe the manner in which the cells are encased one within the other, and the relation they bear to their nuclei, it is necessary to examine them in an earlier stage, and before their walls have begun to be split up into fibres.

The main point distinguishing carcinoma alveolare from carcinoma reticulare and carcinoma simplex seems to be, that, in the former the cells continue to grow, and their walls become adherent to each other, while this progressive development and mutual cohesion do not take place in the delicate cellular globules of the two latter forms of cancer." 54.

*Carcinoma Melanodes.*

M. Müller starts by observing, that melanosis is merely a variety of cancerous degeneration, for it terminates in the same way as carcinoma, and is frequently combined with other species of it.

Carcinoma melanodes is generally lobulated, whether it forms the whole of a morbid growth, or is merely interspersed through the substance of some

* " A short notice of them appeared in an article by Schwann, in Froriep's Not. 1838, Januar. No. 3.

other form of cancer. When it appears in the substance of an organ, it forms masses more or less completely isolated.

"Microscopic examination detects two forms of melanotic structure. In both instances the basis of the structure is formed of a fibrous network, the stroma of melanosis, within the meshes of which the melanoid matter is deposited. This matter is generally composed of cells, filled with yellowish or blackish granules. These cells are, and always continue to be free, never becoming coherent. Their forms are very various. Many, indeed most, are round, oval, or irregular; some are elongated; a few actually caudate, terminating at one or both extremities in a point, or in a fibril. Still more rarely the cells present several points. They are real pigment cells;* some of them are of a palish yellow colour, others darker, while the interior of others is stained of a dark brown by the granular pigment they contain. It was but seldom, and then only with difficulty, that the author succeeded in detecting, in one of the larger cells, a nucleus with its nucleolus, independently of the pigment granules. The diameter of the cells varies greatly; the largest are more than 0.00108 of an English inch in diameter; while smaller ones had a diameter of 0.00105, 0.00095, 0.00073, 0.00045, 0.00039, or even less, of an English inch." 56.

It has not been ascertained whether the increase in number of the pigment cells depends on the production of fresh ones within the parent cell, or whether new cells are formed external to the old ones.

The pigment globules, continues M. Müller, when very small, display that molecular motion common to all very minute parts, even to the globules of the pigmentum nigrum of the eye. The pigment globules are seen not merely within the cavities of the pigment cells, but also strewn between them, and it is only in the case of these free lobules that the molecular motion is observed. The existence of free pigment granules, external to the cells, is perhaps to be attributed to the bursting of the cells and the extravasation of their contents. Many pigment cells are much smaller than others; probably they are young cells which have been set free by the bursting of the older and larger cells, or possibly they may have been formed external to them. Moreover, many cells, and especially many caudate corpuscules, are of so pale a colour, as apparently to be quite destitute of pigment.

In some specimens of melanosis, the author discovered no pigment cells, but all the pigment globules appeared to be free, and dispersed through the meshes of a fibrous tissue. He thinks it probable, that, in these instances, the formative organs of the pigment granules were dissolved, for the latter are always contained in cells.

### *Carcinoma Medullare.*

M. Müller adopts the opinion generally entertained in this country that

---

* A short notice, by the author, of the caudate corpuscules in melanosis appeared in Müller's Archiv. 1837, Heft. v. p. 466, Anmerkung. The author likewise made some observations on the pigment cells of melanoid structures in a postscript to Schwann's third paper, in Froriep's Not. 1838, April.

fungus hæmatodes and medullary sarcoma are only different designations for accidental varieties of the same structure.

The soft cancer, of the consistence of the brain or of the placenta, may have not merely a whitish or yellowish white colour, similar to that of the cerebral substance, or a blood-red hue like the placenta, but it is subject to many other variations of colour; and sometimes the same morbid growth will exhibit all varieties of hue in different parts of its substance.

These fungoid growths are highly vascular, but they present, in addition, a medullary mass composed of globules or other corpuscules, and a tissue made up of delicate fibres, in the meshes of which the medullary portion of the growth is contained.

"When perfectly free from other matters, the medullary part of these growths presents a whitish or greyish white colour. If a portion of fungus medullaris is cut in pieces and squeezed under water, the medullary corpuscules, which are very easily soluble, impart to the water a milky hue, more or less tinged with blood. The intensity of the red colour of fungus medullaris depends on the relative proportion of bloodvessels which it contains; but the bloody patches which are sometimes interspersed through the substance of the structure are in part produced by the effusion of blood into the meshes of its tissue. The brownish hue which the ulcerated surface of fungoid growths presents, is probably produced by decomposed blood.

The external form of fungus medullaris is often lobulated: its appearance when cut or broken varies greatly; sometimes it shews no trace of any definite arrangement of fibres, while, at other times, fibres are indistinctly seen either running parallel to each other, or intersecting each other irregularly, and in some instances displaying a radiated or tufted arrangement. In few cases, however, is this fibrous structure very distinct, for the morbid growth may easily be torn in other directions than in that in which the fibres seem to run; and irregular pieces may often be broken off, though it is not possible to tear off a regular tuft of fibres." 60.

Fungus medullaris usually forms large tumors, but, in some few instances, it presents itself in the shape of a great number of very small ones. It occurs at all ages, and in every organ and vascular tissue. When it appears on the surface of the tubular or flat bones, it receives a slight support from a peculiar skeleton formed of very delicate aciculæ or laminæ of bone, which, proceeding in a radiated manner from the surface of the bone, penetrate into the interior of the soft tumor. Yet Sir A. Cooper successfully amputated a limb affected with a fungous exostosis containing such spicula. If fungus medullaris has its seat in the interior of a bone, not merely does it fill up the cavity of that bone, but induces a state of atrophy in the osseous tissue, and reduces the substance of the bone to a mere shell, so that the slightest cause suffices to produce fracture. It rarely happens that the bone is distended in a spherical manner by medullary fungus in its interior.

The relation of medullary sarcoma to carcinoma simplex is shewn by the fact that, after extirpation of a scirrhous breast fungoid growths not unfrequently follow.

"This affinity is likewise further illustrated by microscopic examination, which shews that many structures comprehended under the generic term of fungus medullaris differ greatly from each other, and have nothing in common but the softness of their texture. Several forms, which present no external differences from others, approach very nearly in structure to the most consistent species of cancer, carcinoma simplex, and contain similar cells or formative globules; the softness of their texture being produced by the presence of a great number of these globules distributed through a very delicate tissue. On the other hand, we have seen that carcinoma reticulare, a form of cancer which presents a very peculiar structure, varies in consistence from that of the hardest scirrhus, to that of fungus medullaris. Further, there are varieties of fungus medullaris, the exterior of which presents nothing peculiar or different from other forms, but which shew a great particularity on microscopic examination, appearing then to be formed in a great measure of caudate or spindle-shaped bodies, or of cells, the development of which has been arrested, while in the intermediate state between cells and fibres. At first, one might be tempted to separate this form from the rest, under the name of carcinoma closteroides; but more extended investigations prove that this division would be unwarrantable, for cases are met with in which, although the medullary part of the morbid structure is composed principally of formative globules without caudate appendages, yet caudate bodies do occur in greater or less number among the round corpuscules. It will hereafter be seen that this variety of internal structure is met with also in the case of innocent albuminous sarcoma, which is sometimes composed of cells, while at other times, it consists of caudate and spindle-shaped corpuscules so arranged as to produce the appearance of an imperfectly fibrous structure." 63.

M. Müller accordingly employs the term fungus medullaris collectively for different forms or stages of development of soft cancer; referring to this genus the following varieties:—

1. Carcinoma medullare, abounding in roundish formative globules which make up the greater part of the medullary mass, though intersected by a delicate fibrous network. These formative globules, M. Müller regards as very similar to those of common cancer, and to those which constitute the grey mass of carcinoma reticulare: a few points, or very minute granules, were often all that could be detected in their interior, but frequently, on making use of a high magnifying power, a nucleus may be seen just as in other forms of carcinoma. The size of these globules is about the same as in common cancer, though it presents great varieties.

2. Carcinoma medullare, with an exceedingly soft cerebriform base, composed of pale, elliptical bodies, without caudate appendages. The author is acquainted with but one specimen, which he can refer to this class: it was a case of cerebriform fungus medullaris of the foot, and of the interior of the tarsal bones. With the exception of the vessels which were distributed to the diseased mass, its substance was almost entirely formed of uniform ellipsoidal corpuscules, which cohered but very slightly with each other. These corpuscules had a very pale hue when looked at under the microscope; they were one-and-a-half or twice as large as the red particles of the blood, and equalled them in breadth. The author in no instance observed a fibril proceeding from these bodies, nor did he ever see a single nucleus or a young cellule in their interior. A few very minute points were all that could be detected by the highest magnifying powers.

3. Carcinoma medullare, with caudate or spindle-shaped corpuscules. Sometimes, on tearing a piece of this kind of fungus medullaris, the torn surface will present a resemblance to a fibrous structure. This appearance is owing to several of the caudate corpuscules being arranged in one direction; as was observed in a case related by Valentin. The author has seen a similar structure in several specimens of fungus medullaris; sometimes interspersed in the midst of round, formative globules, at other times forming the greater part of the growth.

M. Müller goes on to state:—"According to the direction in which the caudate corpuscules are disposed, a radiated appearance is sometimes produced, at other times the structure seems more tufted, while, in other instances the direction of the corpuscules is so various, that the tumor does not display the slightest trace of fibrous texture. It is, indeed, not always easy (even when the caudate bodies are so disposed as to occasion a fibrous appearance) to tear the growth into tufts of fibres, although irregular portions of it may be broken off very readily. Frequently, however, the caudate corpuscules are arranged with great regularity. Their interior presents the appearance already described, namely, it contains either a granular substance without any evident nucleus, or a nucleus with one or more nucleoli may be more or less distinctly seen. These corpuscules are prolonged at one or both sides, and in some rare instances at more than two sides, into fibrils of different length. They are cells, the development of which has been arrested in the stage of transition from cells to fibres."

M. Müller observes that, since so many fibrous tissues in the embryo are formed from caudate cells, there is evidently nothing extraordinary in the occurrence of caudate corpuscules in morbid growths of very different physiological tendencies; in the innocent, as well as in the malignant; and, consequently, no inferences can be drawn from their presence with regard to the character of the structure in which they occur. The only guide, he says, which remains is afforded by the tendency of cancer to interfere with the natural structure of surrounding parts, while those formations which are of a benignant nature leave the neighbouring healthy tissues unaltered. The caudate corpuscules being formed from round cells, the occasional coexistence of round cells with nuclei, and of caudate corpuscules in the same medullary fungus, is not surprising. In all cases of medullary sarcoma fat is present in the shape of fat globules which are free, and not enclosed in cells.

### *Carcinoma Fasciculatum. (Syn. Hyalinum.)*

Some structures ranked as fungus medullaris, though soft, are fibrous in their texture. This is evident on breaking or dividing them.

"If examined under the microscope, they display neither the cellular globules of other varieties of carcinoma, nor the caudate corpuscules which give a fibrous appearance to some forms of fungus medullaris. The fibres often have a tufted arrangement, running in a divergent course from a common centre; in which case the masses may be rent into radiated bundles, the apex of which is directed towards their point of insertion, their base towards the uneven surface of the tumor. Or, some of these tufts of fibres are arranged in one way, some in another, large masses of fibres forming but one tuft, while in other places they are divided into many; and all of these bundles of fibres are intertwined with each other, as is seen on attempting to tear their tissue. In this case, the tumor very frequently forms lobules of various sizes, both externally and in its interior. Between the lobules are membranous septa, from some one of which a tuft of fibres springs, and, after running for some distance, curves over, and is inserted into another septum. These lobulated tumors with a soft fibrous structure often attain a very great size. In some instances, however, there is no distinction of the tumor into lobules, but the whole growth is formed of one large tuft of fibres (having a radiated arrangement), and presents only a slightly uneven surface. These growths are extremely vascular, and their vessels follow the same arrangement as the fibres, observing a penecillous distribution through the interior of the structure, and forming a vascular network on its blood-red ulcerated surface. Occasionally, the substance of the growth is transparent, like jelly." 67.

This circumstance it was that induced M. Müller to name the disease carcinoma hyalinum. But transparency is not a constant character, and the term fasciculatum is therefore preferable. The form of disease seems pretty frequent.

The fibres of carcinoma fasciculatum are extremely pale and transparent, so that it is only by damping the light very much that they can be distinctly seen under the microscope. Their surface is beset here and there with granules, as with an incrustation. M. Müller cannot say whether softness is an invariable character of carcinoma fasciculatum.

He adds :—In organs affected with cancer, fibrous masses may often be observed, which differ greatly from this fasciculated form of carcinoma; while, in the firmness of their substance and in the complete entanglement of the bundles of fibres of which they are composed, they resemble the benignant fibrous tumors of the uterus, and of other parts. The author once saw such masses in the substance of the uterus, in a case of carcinoma uteri. Sometimes the skin covering cancer of the breast becomes thickened, is rendered more dense than natural, and displays, when divided, a similar complicated intertexture of fibres. Lastly, in cases of cancer of the stomach, in addition to the striated appearance which the swollen muscular coat of that organ usually presents when divided, the author has frequently noticed membranous capsules, containing masses made up of bundles of fibres all arranged in one direction.

*Development and Softening of Carcinoma.*

The development of cancer is best studied at present in carcinoma alveolare. This contains within its cells entire generations of younger cells, all of which (as Schwann has shewn to be the case in the early growth of cartilage, and of the chorda dorsalis) are produced from cystoblasts, which, from their large size and dark yellow colour, are easily distinguishable in carcinoma alveolare. The process of development of the other forms of carcinoma cannot be described with the same certainty. On their development, therefore, we need not enter.

In tumors, says M. Müller, with caudate corpuscules, these bodies are evidently formed from cells with nuclei; for in all growths of this class, the author observed, in addition to the caudate or spindle-shaped corpuscules, a few cells with granular contents more or less evident, often with a distinct nucleus. This form of cell obtains in all parts of the growth which have not a distinctly fibrous structure. Moreover, the spheroidal cells pass into the caudate by imperceptible transitions, while the caudate corpuscules become in their turn transformed into fibres, and are themselves the lowest grade of fibrous structure.

Softening and inflammation are the precursors of the ulcerated state of scirrbus. Sometimes they occur first at one part of the tumor, at other times at another; but the statement that they always commence in the interior is quite unfounded. Often, as in the case of carcinoma mammæ, softening begins in the interior, where cavities are frequently found, filled either with a moderately firm or semi-fluid matter. The more consistent matter is the substance of cancer in a state of softening.

"The author ascertained this to be the case in carcinoma reticulare, and he likewise observed, some time since, that the white globules which constitute the peculiar network of that structure not merely accumulate during the progress of the disease, but likewise form a main part of the disorganised mass when softening commences. The softened matter, which resembles pus in its appearance, is either contained within large or small cavities, which, in some instances, communicate with each other; or, in cancer of the mammary gland, it occupies the lactiferous tubes and lymphatic vessels, from the divided cavities of which it exudes on pressure." 71.

In other instances, he continues, the softening and disorganization commence on the surface, as is invariably the case in carcinoma alveolare of the stomach, in which the innermost layer pours out the jelly-like contents of its cells into the stomach. This also takes place sometimes in carcinoma simplex, and in carcinoma reticulare of the mammary gland. The ulcerated surface either shoots forth a cancerous fungus, or destruction and disorganization proceed on the surfuce, unattended by formation of new growths. The latter is often the case in carcinoma of the face; more rarely in carcinoma mammæ. An ulceration with an indurated circumference forms on the surface of the hardened mass, and from this ulceration the different elements of cancer are discharged. M. Müller seems to think it probable, with Henle, that, in all cases of vascular granulations of suppurating surfaces, portions of the real substance of the part enter into the formation of the pus. He observes that the surface of an ulcerated carcinoma presents the same structure as the rest of the morbid growth.

Occasionally, ulcerated carcinoma of the breast cicatrizes a short time before death. According to M. Pouteau, such scirrhi are particularly intractable.

### *Chemical Properties of Carcinoma.*

Cold water extracts from carcinomatous growths, when recent, a small quantity of soluble albumen and osmazome; but by far the greater proportion of their substance is formed of a substance resembling albumen, and insoluble by boiling. Several chemists have asserted that gelatine is a component of carcinoma, but M. Müller is of opinion that it results, at all events in a great degree, from the cellular tissue engaged in it.

Caseine is always found in carcinomatous tumors of the breast, and is probably not to be attributed to milk in the remains of the lactiferous tubes, since M. Boutin Limousineau detected it in carcinoma alveolare pultaceum, and the author obtained it from fungus medullaris of the kidney, as also from many other morbid growths. The existence of caseine in decoctions of carcinoma and of fungus medullaris was proved beyond doubt by several experiments which M. Müller relates, but which we need not particularise.

"With regard to the fat contained in carcinoma, Collard de Martigny speaks of a soft fat in scirrhus of the breast; Wiggers, of fat, containing phosphorus, in fungus medullaris. On the other hand, Gugert detected cholesterine in a fungus medullaris of the eye; Breschet found it in a scirrhus, and Lassaigne in carcinomatous ulcers of the intestine and mesocolon of the horse. Cholesterine is likewise present in many non-carcinomatous pathological structures." 78.

### *Nature of Carcinoma.*

Having gone, almost literally, through the preceding laborious observations of our author, we conclude his account of carcinoma and the present article, by a summary of the results to which his inquiries have led. They are presented in the shape of ten distinct propositions which we shall take, as he offers them, in order.

1. *Carcinoma differs from simple induration not only in its nature, but also in its structure.*

M. Müller thinks there can be no doubt of this, though C. Wenzel laboured to prove that scirrhus and induration are identical, and that carcinoma is merely inflammation in indurated parts. The affinity, however, between scirrhus and fungus medullaris, and the absence of induration in the latter, seem sufficient, of themselves, to upset this notion, were there no other arguments against it. M. V. Walther has ingeniously remarked that induration is the result of a cause which, in occasioning it, has ceased to act—scirrhus the product of a cause still in operation. M. Müller adds:— This opinion is quite supported by minute examination. Not merely does carcinoma simplex, or scirrhus, develop itself without inflammation, but its structure differs from the very first from that of simple induration. Exuded fibrine always has the same appearance, whether it forms false membranes on the surface of organs, or whether it is deposited in their tissue so as to cause induration. Recent exudations do, indeed, contain small globules, but no cellular globules with germs of new cellules.

The translator, however, remarks on this in a note:—

"It does appear doubtful whether we are warranted in laying down so positively as is here done, by Professor Müller, the distinction between carcinoma and induration. Dr. Henle remarks on this subject, that fibrinous exudations contain not merely globules, but also cells, which, though not furnished with germs of young cellules, contain the characteristic nuclei, and at an early period become elongated, and transformed into fibres of cellular tissue, such as constitute the cicatrices of ulcers, &c." 80.

2. *Carcinoma differs also in its nature from ulcerations of indurated parts.*

On this point we feel disposed to cite M. Müller's criticism upon Andral, for it exposes, we think justly, a cardinal error of the French school. It is astonishing, indeed, considering their merits as pathologists, how inaccurate the ideas of the French are on the subject of cancerous diseases.

"Wenzel denies that there is any important difference between ulcerations of indurated parts and ulcerated carcinoma; and Andral even asserts, that the products of all morbid secreting and nutritive processes become cancerous in the stage at which they pass into progressively increasing ulceration. This mode of expression is altogether metaphorical, and belongs, like the word inflammation, entirely to the infancy of science: it does but indicate the common result of very different changes of structure, not one disease *sui generis;* and, did it not emanate from a very distinguished and meritorious writer, it might be passed over without further comment. This confounding of diseases, definite in character, and destructive from their very commencement, with others which prove fatal only by loss of the fluids, and by disturbance of sanguification, seems to the author to be as little of an advance in science, as is that revolution which Andral has sought to bring about in the doctrine of inflammation by his notions about hyperæmia. Most ulcers of a non-carcinomatous nature are ulcers in indurated parts, for exudations often take place in the circumference of abscesses and of ulcers, and thus cause induration of the surrounding parts. Henle's investigations, indeed, have shewn that the granulations of ulcerated surfaces of all sorts are composed of cells resembling those in the tissues of the embryo. This, cannot however, be adduced in proof of the similarity of carcinoma and of ulcers in indurated parts, for most benignant tumors are also made up of embryonic cells. Rather, may we say, that the nature of both morbid structures is physiologically different in regard to the productive and destructive powers which each possesses. It is only in carcinoma that the most dissimilar tissues of parts become transformed into tissues identical with those which form the morbid growth." 81.

3. *Carcinoma is no heterologous structure, and the minutest elements of its tissue do not differ in any important respect from the constituents of benignant growths, and of the primitive tissues of the embryo.*

This seems a startling assertion, and must take many by surprise, as well as, perhaps, occasion some mortification to those who have confided in the advances of morbid anatomy in the department of the morbid growths. M. Müller, however, supports his proposition thus;—

"The elements of carcinoma are nuclei, cells, caudate corpuscules which are developed from cells, and fibres which are formed from caudate corpuscules. No other elements occur in benignant tumors. The gelatine-yielding enchondroma and the albuminous sarcoma consist of cells; sarcoma with caudate corpuscules contains the same elements as the corresponding form of fungus medullaris. The gelatine-yielding cellulo-fibrous tumor, the gelatine-yielding tendino-fibrous or desmoid tumor, and the albumino-fibrous tumor, are all, like carcinoma fasciculatum, composed of fibres. The pigment cells of melanosis are repetitions of healthy pigment cells. The peculiar appearance of the white corpuscules in carcinoma reticulatum, and their reticulated arrangement, occurring as they do in but one form of carcinoma, do not warrant us in founding thereon any theory of the heterology of cancer." 81.

The translator remarks that Henle objects to this argument of Müller's. He doubts the adequacy of microscopic and chemical investigations, and thinks that the development of cells, apparently resembling each other, into tissues physiologically dissimilar, must be owing to some difference which we cannot appreciate in their component organic matter. However that may be, and the supposition is a reasonable one, it is clear that we have no right, at the present time, to assert the existence of structural differences, when our most laborious researches can prove none, and the plain confession of ignorance and uncertainty is better than the assumption of knowledge.

4. *Neither does Carcinoma (independently of the sanies) possess any peculiar chemical constituents.*

The materials observed in carcinoma are albumen, gelatine, caseine, a matter resembling salivary matter, and fat, one form of which is cholesterine. These, however, are all contained in many other non-carcinomatous growths.

5. *The peculiar nature of the destructive and productive activity of carcinoma does, however, determine in it general anatomical characters which may, in most cases, be distinguished with the naked eye.*

Among these, says M. Müller, may be reckoned the removal and dissolution of the elements of the affected organs; the transformation of muscles, tendons, nerves, and membranes into the same new mass; the peculiar grouping of the elements of carcinomatous growths, the cerebriform softness of medullary sarcoma, the reticulum of carcinoma reticulare, the production of pigment with the destructive development of melanosis, the peculiar structure of carcinoma alveolare with the same destructive tendency, and others which might be mentioned. Thus may fungus medullaris with caudate corpuscules be distinguished from the corresponding benignant sarcoma, for the latter leaves untouched the different structures in or near the affected organ. A benignant sarcoma of the conjunctiva, though as large as the fist, left the globe of the eye unaltered; while a much smaller carcinomatous growth in the same neighbourhood would occasion the nerves, muscles, and tunics of the eye to swell, and to develop the peculiar morbid structure.

This brings us back, we fancy, to where we were before the microscope and chemistry were applied to the analysis of carcinoma. We must study the general characters, and make ourselves as well acquainted with them as we can.

6. *The development of carcinoma is the result of a diseased state of the vegetative process; which, whether general or local in the first instance, always tends to involve the whole constitution.*

Unfortunately this needs no proof.

7. *Although in most instances a general disposition to carcinoma exists from the time of its commencement as a local disease, yet it must be owned that a local disposition may give rise to carcinoma, which may afterwards contaminate the whole constitution; and this local disposition has been proved to be, in some instances, the result of certain external agents.*

There is little doubt of the correctness of this statement—the difficulty is to apply it to individual cases. M. Müller is convinced that, in some very rare cases, true carcinoma has been cured by extirpation. He refers to some instances of reputed cure. But he justly observes, that it is not quite sure in these cases that the disease was carcinoma, for innocent tumors, when incompletely extirpated, very readily return. Cases which have been operated on once only, and then with perfect success, while the morbid growths have not been anatomically examined, must be left quite out of consideration, since diseases of all sorts have been extirpated for scirrhus and cancer. The following seem unobjectionable.

"The author's friend, Professor Pockels, says that once, from among many melancholy cases, he cured a true carcinoma of the breast by extirpation. In this instance, the structure of the tumor was considered by Professor Pockels to be identical with that already described as carcinoma reticulare. Professor Jüngken extirpated, from the orbit of a young woman, a compound carcinoma, partly made up of carcinoma reticulare, partly of carcinoma melanodes: two years have now elapsed since this operation, and the patient continues in perfect health; while other precisely similar cases have had a fatal termination. A carcinoma reticulare of the female breast, which was extirpated by M. v. Graefe, did not return till after the lapse of five years. The author examined the degeneration as it appeared in the breast last affected, and found it to be carcinoma reticulare." 85.

He alludes, with scepticism, to several reputed cures of fungus medullaris. The cases on which most dependence can be placed are those in which fungus medullaris of the globe of the eye retroceded, without any operation having been performed, before the cornea had become perforated. Such cases, indeed, are very rare.

M. Müller refers to chimney-sweepers' cancer, as affording the best instance of a disposition to carcinoma at first merely local, though afterwards tending to become general. He mentions, too, cancerous affections of the face and lip, which are sometimes cured by local treatment, and the malignity of which, must, in those instances, have consisted in a merely local disposition. He adds, that, it is very difficult, and not always possible, to distinguish, by any peculiarity of structure, cancer of the skin from neglected or mismanaged ulcers of that texture. Here the characteristic forms of cancer are very rare; in one only of many instances was the disease found to be carcinoma reticulare. In no part of the pathology of cancer are our notions more unsettled than in this instance. The mere malignity of a sore is insufficient to stamp on it the carcinomatous character, for then must herpes rodens be cancer. To the idea of a local cancerous affection belong the development of the disease from carcinomatous tubercles; the property, when left to itself, of destroying, without intermission, the structure of all tissues with which it meets, whether muscles, mucous membranes, or bones (the last without the phenomena of ordinary caries or necrosis); and, lastly, the property of giving rise to any of the various forms of carcinomatous degeneration. The diagnosis is still more difficult, if a non-carcinomatous ulceration of the skin should, from the supervention of a carcinomatous disposition, become converted into cancer, for then we lose the guide afforded by the origin of the disease from a carcinomatous tubercle.

M. Müller admits the impossibility of comprehending the mode in which the carcinomatous dyscrasia becomes developed from a merely local disposition. It can, however, he thinks, be easily understood, that, when once cells with a productive tendency have been formed, the reception of the germinal nuclei into the circulation may determine their distribution to some part predisposed to receive them, and may thus give rise to the formation of secondary tumors. He is not clear what importance is due to the appearance of masses of fungus medullaris within the cavities of large vessels, especially veins.

8. *Some tumors which by nature are not carcinomatous, and part of the*

*character of which it is to remain local, may, under certain circumstances, originate the local disposition to cancer.*

"Aneurism by anastomosis, and nævi materni, must be referred to this class; for. M. v. Walther has shewn that, under long-continued irritation from internal or external causes, they may be converted into fungoid growths possessed of properties similar to those of cancerous tumors. They are less susceptible than other parts of simple inflammation and its consequences." 90.

9. *Many structures differing from carcinoma have, on the other hand, even though repeatedly mismanaged, no inclination to assume the cancerous disposition; or, perhaps, they may be more correctly said to have no greater disposition to pass into the carcinomatous state, than is possessed by many other healthy tissues.*

To this class may be referred the simple fatty tumor, and the tendino-fibrous or desmoid tumor. M. Müller's observations would lead him to say, that the carcinomatous tendency is not greater in enchondroma, cholesteatoma, and in albuminous sarcoma and osteosarcoma (cellular sarcoma, sarcoma with caudate corpuscules, and fibrous sarcoma). Irritation and partial excision determine, indeed, an increased growth of these tumors; but if perfectly extirpated they cease to be reproduced, and if at any time they exert an injurious influence on the constitution, it is merely by the loss of fluids which they occasion.

10. *Each form of cancer occurs in persons of all ages, and in all organs, but some organs appear to be especially liable to carcinoma at certain periods of life.*

"It has already been long known that fungus medullaris occurs indifferently at all periods of life. Many instances have also occurred to the author which prove that carcinoma reticulare, the most common form of cancerous degeneration in the breast of females advanced in life, is a disease incidental to all ages. The author frequently saw it in the orbit of children, where he has also often met with carcinoma medullare. It is, therefore, incorrect to say, that the ordinary cancer of the breast holds, as a distinct form of cancer, any peculiar ætiological relation to the climacteric years in women; such a relation has reference to the organ attacked, not to the disease by which it is affected. The breast is, like the uterus, more frequently attacked by carcinoma at this period; but this form of cancer occurs at all periods of life.

The assertion has often been made, though without foundation, that certain tissues are affected by peculiar forms of carcinoma. It has, indeed, been allowed, that fungus medullaris may occur in all organs and tissues. In the orbit, it attacks indifferently all parts of the eye. Hence it is evident that the question, whether in this or the other case, the affection began in the optic nerve, or in the choroid coat, or in some other part of the eye, arises from an erroneous conception of the subject. It is true that in some cases the affection may be particularly well marked in this or in the other part; but there are instances in which the same degeneration becomes simultaneously evident in the muscles of the eye, in the sclerotic, choroid coat, optic nerve, and crystalline lens. Proofs of this are afforded by the numerous writings upon this subject, and the author can substantiate them from the results of his own observations on morbid growths, extirpated by M. Jüngken. Medullary sarcoma of the bones of the cranium, of the dura mater, and of the brain, sometimes originates in one of these parts, sometimes simultaneously in them all.

Carcinoma simplex, or scirrhus, has been said to be peculiar to glandular structures. It is well known that it may appear in the bones at the same time with scirrhus in the breast, as well as after its extirpation." 92.

In a case of extremely hard scirrhus of the breast, the author observed formative globules precisely similar in some very hard tumors which grew from the cancellous structure of the ribs. In another instance, carcinoma fasciculatum was developed in the mammary gland, in the orbit, and in a cancerous fungus of the skin. Carcinoma alveolare and reticulare have likewise an universal distribution, and fungoid melanosis notoriously has so.

This completes our able author's account of Carcinoma. We believe that his observations are new to the majority of English readers, and we have therefore given them very much at length. However satisfactorily they may display his industry and his pathological sagacity, we fear they as satisfactorily shew how little microscopical and chemical investigations have hitherto done in clearing up the mystery that surrounds the malignant morbid growths. We cannot conscientiously say that they have added much to our substantial knowledge on the subject. But we are not to despair. Minute researches always leave us rather wiser than they found us, and often when discovery seems as remote as ever, it is actually impending. There ought to be no bounds to our exactness save what nature has made impassable.

We shall return, in our next number to M. Müller's work, and to the non-carcinomatous growths.

